# Tumor Necrosis Factor Receptors and C-C Chemokine Receptor-2 Positive Cells Play an Important Role in the Intraerythrocytic Death and Clearance of *Babesia microti*

**DOI:** 10.3390/pathogens13100858

**Published:** 2024-10-01

**Authors:** Dana G. Mordue, Adiya S. Katseff, Andrew J. Galeota, Synthia J. Hale, Shaaf Rezaee, Ilana Schwartz, Mariya Sambir, Paul M. Arnaboldi

**Affiliations:** 1 Department of Pathology, Microbiology, and Immunology, New York Medical College, Valhalla, NY 10595, USAagaleota@student.touro.edu (A.J.G.); ilanaschwartz@brandeis.edu (I.S.);; 2 Touro College of Dental Medicine, Hawthorne, NY 10532, USA; 3 Biopeptides Corp, Ridgefield, CT 06877, USA; 4 Lovelace Biomedical Research Institute, Albuquerque, NM 87108, USA

**Keywords:** *Babesia* species, host response, babesiosis, tick-transmitted infections

## Abstract

*Babesia microti* is an Apicomplexan parasite that infects erythrocytes and causes the tick-transmitted infection, babesiosis. *B. microti* can cause a wide variety of clinical manifestations ranging from asymptomatic to severe infection and death. Some risk factors for severe disease are well-defined, an immune compromised state, age greater than 50, and asplenia. However, increasing cases of severe disease and hospitalization in otherwise healthy individuals suggests that there are unknown risk factors. The immunopathology of babesiosis is poorly described. CD4+ T cells and the spleen both play a critical role in parasite clearance, but few other factors have been found that significantly impact the course of disease. Here, we evaluated the role of several immune mediators in *B. microti* infection. Mice lacking TNF receptors 1 and 2, the receptors for TNFα and LTα, had a higher peak parasitemia, reduced parasite killing in infected red blood cells (iRBCs), and delayed parasite clearance compared to control mice. Mice lacking CCR2, a chemokine receptor involved in the recruitment of inflammatory monocytes, and mice lacking NADPH oxidase, which generates superoxide radicals, demonstrated reduced parasite killing but had little effect on the course of parasitemia. These results suggest that TNFR-mediated responses play an important role in limiting parasite growth, the death of parasites in iRBCs, and the clearance of iRBCs, and that the parasite killing in iRBCs is being primarily mediated by ROS and inflammatory monocytes/macrophages. By identifying factors involved in parasite killing and clearance, we can begin to identify additional risk factors for severe infection and newer therapeutic interventions.

## 1. Introduction

*Babesia microti* is an intraerythrocytic Apicomplexan parasite that is the most important causative agent of human babesiosis in the US [1]. The parasite is transmitted by the bite of infected *Ixodes scapularis* ticks, the same ticks that transmit *Borrelia burgdorferi*, the causative agent of Lyme disease. *B. burgdorferi* and *B. microti* are often co-pathogens in endemic areas. The parasite can also be transmitted vertically from mother to newborn [2] as well as by transfusion with infected RBCs [3,4]. In otherwise healthy individuals, less than 50 years of age, tick-transmitted infection frequently causes only a mild disease course or can be asymptomatic, likely often going undiagnosed. In individuals greater than 50 years of age, asplenic individuals, or those with a compromised immune system, *B. microti* can cause a severe infection requiring hospitalization and can be fatal [1,5,6]. Asplenia is a particularly significant risk factor for fatal disease. Hospitalization rates vary from 16.0% for those who were 10–19 years old to 72.6% of those >80 years of age in patients with symptomatic babesiosis that prompted a medical visit during a five-year period from 2011 to 2015 [7]. Transfusion recipients tend to have a more severe disease course, as transfusion recipient populations tend to be enriched for individuals with risk factors for severe infection (advanced age, compromised immune status). Transfusion-transmitted babesiosis is one of the most common causes of transfusion-transmitted infections and of death due to microbial contamination of transfused blood in the US [3,4,6].

As with other tick-transmitted diseases, the incidence of babesiosis has been increasing steadily, with the reported number of cases more than doubling between 2011 and 2019 [8]. Despite the risks associated with babesiosis, much about the pathophysiology of the disease is poorly understood. In a previous study, we developed a mouse model of babesiosis where we followed the progression of parasitemia by evaluating blood smears for infected red blood cells (iRBCs) containing healthy single parasites, healthy multiple parasites, or a phenotype called a ‘crisis form’ which is indicative of intraerythrocytic death of parasites [9]. Using this model, we demonstrated that genotypic differences in different strains of mice led to differences in the level of parasitemia that developed during infection, but that ultimately parasitemia was cleared in immunologically intact mice by day 21. We confirmed prior data demonstrating that CD4+ T cells are required for clearance of the parasite during infection, as absence of these cells in CD4-/- mice resulted in the development of long-term persistent parasitemia. We also demonstrated that B cells, IFNγ, TLR signaling through the MyD88 adaptor protein, and iNOS2 production of NO were dispensable for the control of infection, though clearance of the parasite was moderately delayed in mice lacking B cells and IFNγ-/- mice [9].

The immunological factors associated with protection from babesiosis remain undefined. Without a complete understanding of what is necessary to protect from infection, we will continue to be unable to identify some of the risk factors that predispose to severe disease. While severe babesiosis has some well-defined risk factors (described above), which are shared for a large number of infectious diseases, there are clearly factors that we do not know. This is highlighted by the hospitalization of younger individuals with no defined risk factors [7]. In addition, treatment failure is common in babesiosis, particularly in immunocompromised patients [10,11,12,13]. Understanding the underlying immunopathology of this infection will allow for the identification of new targets for therapeutic intervention in severe babesiosis, as well as identifying additional biomarkers that could be prognostic for the development of severe disease. In the present study, we evaluate the role of immunological factors in the intraerythrocytic killing of *B. microti* during infection as well as clearance of iRBCs.

## 2. Methods and Materials

### 2.1. Mice

All work with animals was conducted under approval by the New York Medical College (NYMC) Institutional Animal Care and Use Committee. We utilized the following mice from the Jackson laboratories (Bar Harbor, ME): C57BL/6J, B6N.129S2-Ncf1^tm1Shl^/J (p47^phox-/-^); B6.129S-*Tnfrsf1b^tm1Imx^ Tnfrsf1a^tm1Imx^*/J (p55/p75KO, TNFR1a/1b-/- mice); and B6.129S4-*Ccr2^tm1Ifc^*/J (CCR2-/-). CD-1 mice were obtained from Charles River Laboratories and were utilized solely for recovering parasites from cryopreservation. Mice were utilized between 6 and 10 weeks of age. Breeding pairs of Macrophages Insensitive to IFNγ (MIIG) mice [14] were generously provided by Michael B. Jordan, MD of the Cincinnati Children’s Hospital, and were bred for experiments in the NYMC Department of Comparative Medicine. MIIG mice were utilized between 8 and 20 weeks of age for experiments.

### 2.2. Parasites and Infection Model

For all experiments, we utilized the Gray strain of *Babesia microti*. This strain, which we obtained from the American Type Culture Collection (ATCC 30221) in 2013, was originally derived from the infection of a previously healthy middle-aged woman on Nantucket Island in Massachusetts in 1970 [15]. Parasites were stored in frozen whole blood in Alsever’s Solution with 30% glycerol as per ATCC guidelines. Parasites were recovered by injecting cryopreserved blood intraperitoneally into CD-1 mice. Blood was collected at the height of parasitemia (day 6–7 post-infection) and passed to new mice by IP injection (100 μL) (approximately 1 × 10^7^ iRBCs). Cryopreserved parasites were recovered by passage through 2 rounds of mice before using them for experiments. For experiments, blood was obtained from four to five mice by cardiac puncture and pooled. Moreover, 100 μL of blood was injected into C57BL/6 control mice and indicated experimental mice by IP injection to guarantee that both groups of mice received an identical inoculum. A group of control mice was utilized in every experiment, and results were always compared between the experimental group and control group within each experiment.

### 2.3. Evaluation of Parasitemia

**Figure 1:**
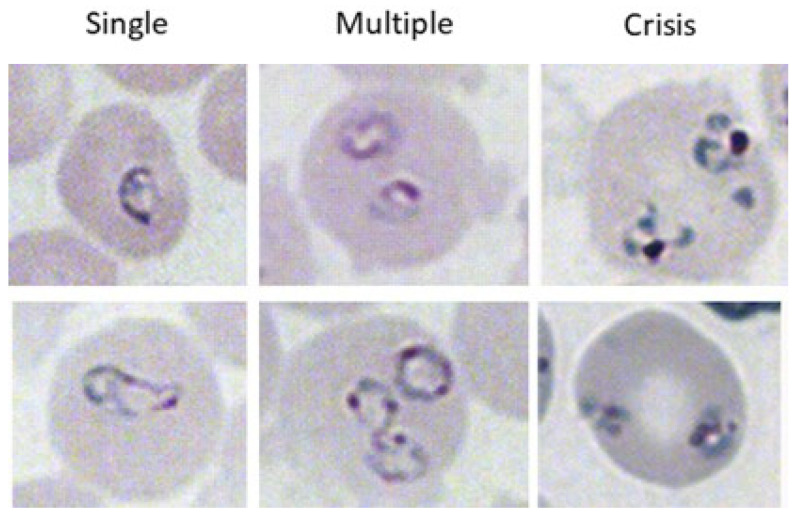
Different phenotypes of *B. microti* within iRBCs. iRBCs were evaluated for single parasites (**left** column), multiple parasites (**center** column), or parasites in crisis form (**right** column). The top and bottom images are two representative images of each parasite form.

Blood was collected at the indicated timepoints by tail snip. Blood smears were immediately created, and slides were allowed to dry. Slides were fixed with ice cold 100% methanol and stained with Giemsa, following the manufacturer’s instructions (Richard Allan Scientific, Kalamazoo, MI, USA). The percentage of iRBCs containing single organisms (Figure 1, left), multiple organisms (Figure 1, center), or organisms in crisis form (Figure 1, right) were calculated by 3 counts of 100 RBCs in 3–5 different visual fields at 100× magnification under brightfield illumination on a microscope. The number of iRBCs containing each type of parasite was averaged. All parasite counts were performed by two individuals: an initial blinded observer and then a validation count. Data are reported as the number of iRBCs containing single, multiple, or crisis-form parasites ± standard deviation.

### 2.4. D89E

A plasmid (peF-BOS-EX) containing the gene for the D89E mutant of Milk Fat globulin-EGF-factor 8 (MGF-E8) was generously provided by Dr. Shigekazu Nagata, Ph.D., of Osaka University [16,17]. The plasmid was transfected into 298T cells, and the flag-tagged protein was purified using previously published methods [18]. Moreover, 10 μg/mL of D89E was administered by IV injection into the retro-orbital plexus of mice 24 hrs prior to infection with *B. microti* and every 7 days for the duration of the experiment, using a modification of a previously published protocol [18].

### 2.5. Statistical Analysis

Statistical differences were only evaluated between control (C57BL/6) and experimental mice for percent iRBCs containing single parasites, percent iRBCs containing multiple parasites, and percent iRBCs containing crisis-form parasites. Each condition only contained two groups: control (C57BL/6) and experimental. Within-group comparisons were not made between single, multiple, and crisis-form parasites, nor was the effect of time on parasitemia assessed statistically. Comparisons were made between groups at each timepoint using the multiple unpaired *t*-test feature of PRISM 10 (Graphpad, Boston, MA, USA), using the Holm–Šidák method with the following parameters: individual variance for each row (timepoint), a set *p*-value threshold for significance of *p* < 0.05. The adjusted *p*-value for multiple tests is reported. Where possible in the text, the actual *p*-value is reported. The maximum number of digits returned was 4, so any value below 4 digits is reported as *p* < 0.0001.

## 3. Results

### 3.1. TNFα Plays a Key Role in Controlling and Clearing B. microti Infection

**Figure 2:**
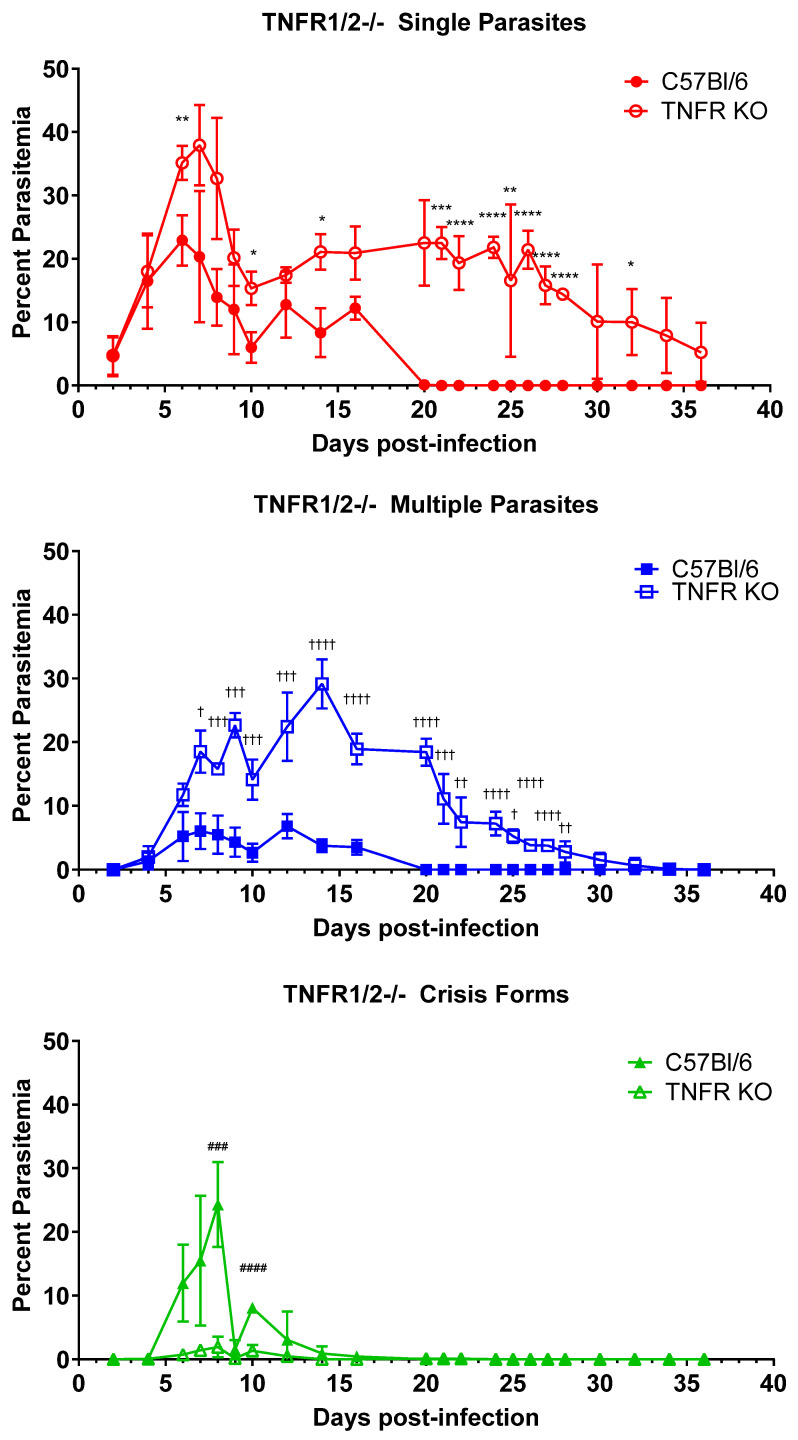
Parasite burden and persistence are increased, and intraerythrocytic parasite killing is reduced in mice lacking TNFR1 and 2. The percentage of RBCs containing single parasites (circles), multiple parasites (squares), or parasites in crisis form (triangles) were enumerated every other day from day 3 post-infection to day 36 post-infection in TNFR1a/1b-/- mice (closed symbols) and C57BL/6 (intact control, open symbols) mice. Levels of single, multiple, or crisis-form parasites were compared between TNFR1a/1b-/- mice and C57BL/6 mice using the multiple *t*-test in PRISM 10; adjusted *p*-values are reported. * *p* < 0.05, ** *p* < 0.01, *** *p* < 0.001, **** *p* < 0.0001 between TNFR1a/1b-/- and C57BL/6 single parasites, † *p* < 0.05, †† *p* < 0.01, ††† *p* < 0.001, †††† *p* < 0.0001 between TNFR1a/1b-/- and C57BL/6 multiple parasites ### *p* < 0.001, #### *p* < 0.0001 between TNFR1a/1b-/- and C57BL/6 crisis-form parasites. *n* = 4–5 mice per group. The C57BL/6 mice in this figure are the same as in Figure 5, as both groups of knock-out mice were infected in parallel and referenced to the same control group.

We previously showed that IFNγ plays a limited or redundant role in the control and clearance of *B. microti* parasitemia. IFNγ-/- mice had a higher peak parasitemia and a modest delay in parasite clearance compared to control mice [9]. As TNFα also plays an important role in the activation of innate immune cells, we sought to assess the role for TNFα in controlling and clearing parasitemia. We infected mice deficient in TNFR1a and TNFR1b, the receptors for TNFα and LTα (TNFβ) [19,20], with *B. microti* and compared the response to immunologically intact C57BL/6 mice (Figure 2). For C57BL/6 mice, the percentage of iRBCs parasitized with a single *B. microti* organism peaked on day 6 p.i. but was significantly lower than that of TNFR1a/1b-/- mice (22.9% ± 4.0 vs. 35.2% ± 2.7 C57BL/6 vs. TNFR1a/1b-/-, *p* = 0.018). Peak parasitemia for single parasites occurred one day later in TNFR1a/1b-/- mice, on day 7 p.i. (20.3% ± 10.4 vs. 37.9% ± 6.3, C57BL/6 vs. TNFR1a/1b-/-, *p* = 0.14). Similarly, the timing and magnitude of peak parasitemia for iRBCs containing multiple *B. microti* parasites differed between C57BL/6 mice and TNFR1a/1b-/- mice, occurring on day 12 in the C57BL/6 mice (6.8% ± 1.9 vs. 22.4% ± 5.4, C57BL/6 vs. TNFR1/2-/-, *p* = 0.006) and on day 14 in TNFR1a/1b-/- mice (3.7% ± 1.0 vs. 29.1% ± 3.9, C57BL/6 vs. TNFR1a/1b-/-, *p* = 0.0003). Crisis-form development was correspondingly reduced in TNFR1a/1b-/- mice compared to C57BL/6 mice (24.3% ± 6.7 vs. 1.9% ± 1.7, C57BL/6 vs. TNFR1a/1b-/-, day 10 p.i., *p* = 0.003). Furthermore, TNFR1a/1b-/- mice developed a persistent parasitemia that lasted beyond the 7 weeks the experiment was carried out for. Blood smears were not fully evaluated until after the experiment had been terminated. C57BL/6 mice cleared the infection by day 21, when parasites were no longer observed in blood smears. iRBCs parasitized with single *B. microti* organisms continue to be observed in blood smears at the final timepoint (day 36) in TNFR1a/1b-/- mice, while multiple *B. microti* parasitized iRBCs were observed through day 34 p.i. The decline in parasitemia over time trended towards eventual clearance. The duration of parasitemia and the magnitude of the difference in peak parasitemia were greater for TNFR1a/1b-/- mice compared to control than were observed for IFNγ-/- mice compared to controls [9], suggesting that TNF receptor agonists play a greater role in parasite control and clearance than does IFNγ. TNFR deficiency remains to this point the only immunologic factor whose loss affects all three parameters examined (peak parasitemia, crisis-form development, and parasite clearance). At this time, we cannot definitively attribute the observed effects to TNFα as the receptors also bind LTα. However, this would be an unconventional role for LTα. These data suggest that one of these factors plays an important role in limiting parasite growth and clearing the infection.

### 3.2. CCR2 Mobilization of Cells Impacts Intraerythrocytic Death of B. microti Parasites but Not Parasite Clearance

**Figure 3:**
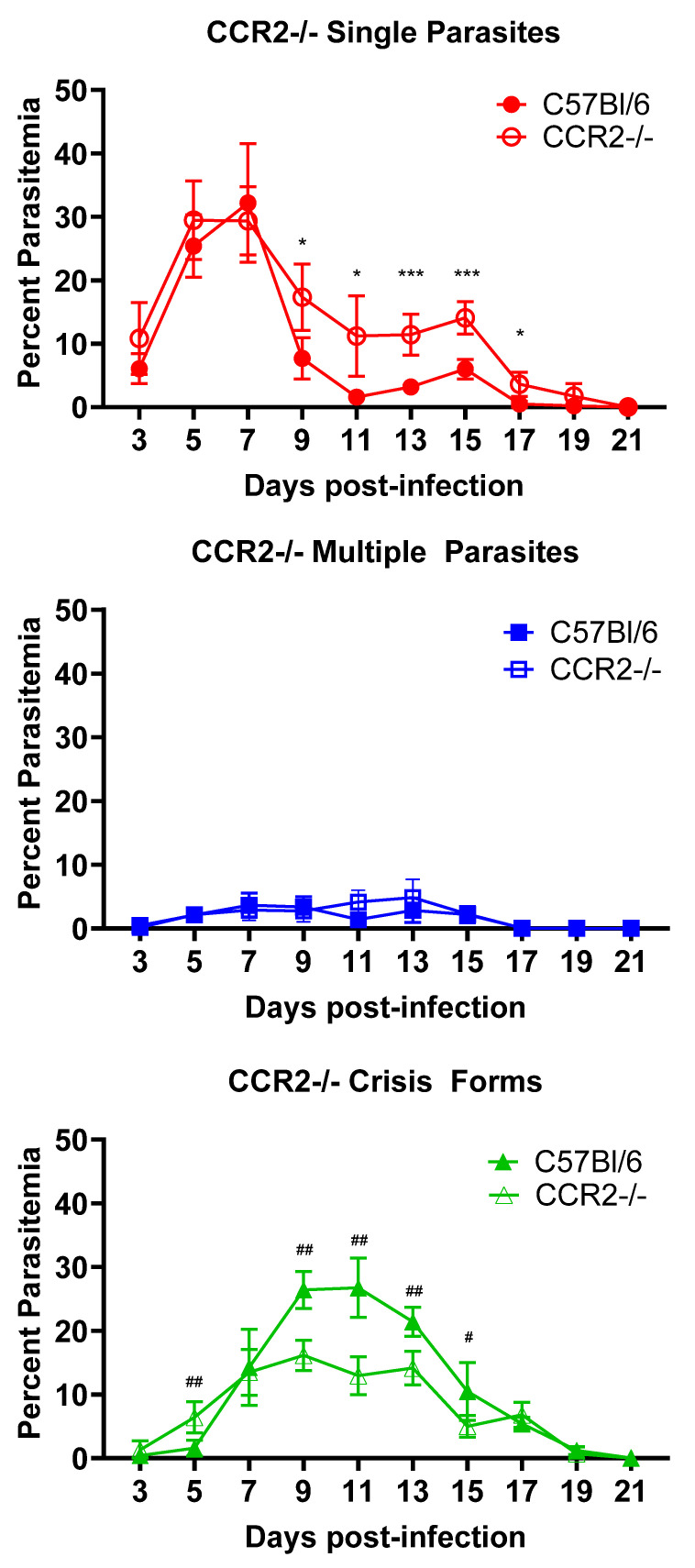
Reduced crisis-form development but not parasite clearance in CCR2-/- mice compared to C57BL/6 mice over the course of infection with *B. microti*. The percentage of RBCs containing single parasites (circles), multiple parasites (squares), or parasites in crisis form (triangles) were enumerated every other day from day 3 post-infection to day 21 post-infection in CCR2-/- (closed symbols) and C57BL/6 (intact control, open symbols) mice. Parasite clearance was observed in all mice at day 21. Levels of single, multiple, or crisis-form parasites were compared between CCR2-/- and C57BL/6 mice using the multiple *t*-test in PRISM 10; adjusted *p*-values are reported. An *p* < 0.05 was considered significant. # *p* < 0.05, ## *p* < 0.01 between CCR2-/- and C57BL/6 crisis forms, and * *p* < 0.05, *** *p* < 0.001 between CCR2-/- and C57BL/6 single parasites. *n* = 5 mice per group.

We tested a hypothesis that inflammatory monocytes contribute to the control and clearance of *B. microti* parasites across infection. For this, we utilized CCR2-/- mice, which have significantly reduced mobilization of inflammatory monocytes from the bone marrow [21]. C57BL/6 mice were used as an intact control. As seen in Figure 3, neither total peak parasitemia nor duration of parasitemia were affected by the lack of CCR2. Similar peak parasitemia was observed on day 7 post-infection in both groups of mice [Single parasites (32.2% ± 9.3 vs. 29.4% ± 5.4., C57BL/6 vs. CCR2-/-, n.s.), Multiple parasites (3.7% ± 1.9 vs. 2.9% ± 1.6., C57BL/6 vs. CCR2-/-, n.s.)]. Parasites were cleared by day 21 in both strains. However, when the RBCs were analyzed for healthy vs. crisis-form parasites, differences emerged. Crisis-form parasites formed slightly earlier in CCR2-/- mice compared to C57BL/6 (6.5% ± 2.4 vs. 1.6% ± 1.2, CCR2-/- vs. C57BL/6, day 5 p.i., *p* = 0.007). However, crisis forms were significantly reduced in CCR2-/- as compared to C57BL/6 mice on day 9 p.i. (16.1% ± 2.4 vs. 26.4% ± 2.9, CCR2-/- vs. C57BL/6 *p* = 0.001), day 11 p.i. (12.9% ± 3.0 vs. 26.8 ± 4.7, CCR2-/- vs. C57BL/6 *p* = 0.001), day 13 p.i. (14.1% ± 2.6 vs. 21.4 ± 2.3, CCR2-/- vs. C57BL/6 *p* = 0.002), and day 15 p.i. (5.0% ± 1.7 vs. 10.5 ± 4.5, CCR2-/- vs. C57BL/6 *p* = 0.037). A corresponding significant increase in the number of single healthy *B. microti* parasites was observed on days 9–17 in CCR2-/- mice compared to C57BL/6 [Day 9: 17.3% ± 5.2 vs. 7.7% ± 3.3. *p* = 0.012; Day 11: 11.2% ± 6.3 vs. 1.6% ± 0.9 *p* = 0.010; Day 13: 11.4% ± 3.2 vs. 3.2% ± 0.2 *p*= 0.0009; Day 15: 14.1% ± 2.5 vs. 6.0% ± 1.5 *p* = 0.0009; Day 17 3.6% ± 1.9 vs. 0.5% ± 0.5 *p* = 0.01; CCR2-/- mice vs. WT mice]. RBCs containing multiple parasites were similar between both groups at all timepoints. These data suggest that CCR2+ cells, likely inflammatory monocytes, play a role in the intraerythrocytic death of the parasite but are not required in the overall control of total parasitized RBCs or clearance of iRBCs. It is important to note that the spleen of mice harbors a pool of inflammatory monocytes, which could be mobilized in the absence of CCR2 as *B. microti*-infected blood passes through the spleen, somewhat mitigating the effects of CCR2 deficiency in these mice. This is being further investigated. These data support a role for distinct immunological mechanisms in the intraerythrocytic death of *B. microti* and its clearance.

### 3.3. IFNγ Activation of Macrophages Is Not Required for Killing or Clearance of Babesia microti

**Figure 4:**
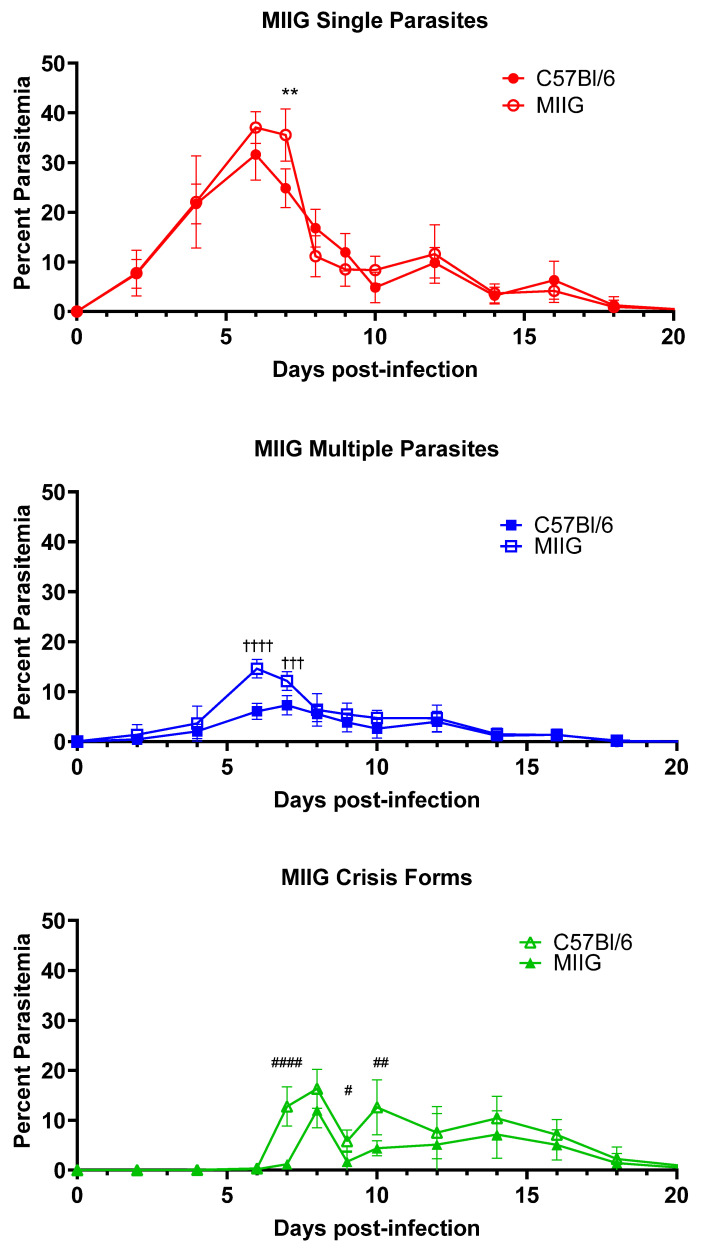
Increased multiple parasite parasitemia and reduced crisis-form development in MIIG mice compared to C57BL/6 mice over the course of infection with *B. microti*. The percentage of RBCs containing single parasites (circles), multiple parasites (squares), or parasites in crisis form (triangles) were enumerated every other day from day 3 post-infection to day 21 post-infection in MIIG mice (closed symbols) and C57BL/6 mice (intact control, open symbols). Parasite clearance was observed in all mice on day 21. Levels of single, multiple, or crisis-form parasites were compared between MIIG mice and C57BL/6 mice using the multiple *t*-test in PRISM 10; adjusted *p*-values are reported. An *p* < 0.05 was considered significant. ** *p* < 0.01 between MIIG and C57BL/6 single parasite, # *p* < 0.05, ## *p* < 0.01, and #### *p* < 0.0001 between MIIG and C57BL/6 crisis-form parasites, and ††† *p* < 0.01, †††† *p* < 0.0001 between MIIG and C57BL/6 multiple parasites. *n* = 10 mice per group.

Given the requirement of CD4+ T cells and the central role of the spleen in the clearance of *B. microti*, we sought to clarify the role of IFNγ secreted by CD4+ T cells on activated splenic macrophages in optimal clearance of the infection. To test this hypothesis, we infected Macrophages Insensitive to IFNγ (MIIG) mice with *B. microti* and assessed killing and clearance of the parasite (Figure 4). MIIG mice have a dominant negative IFNγR1 expressed in cells expressing CD68, which includes various mononuclear phagocytes/macrophages and dendritic cells, though low expression levels have been suggested in other cell types [14]. In these mice, macrophages do not respond to IFNγ stimulation, and DC responses to the cytokine are reduced. Infection of MIIG mice with *B. microti* resulted in a mild increase in peak parasitemia of iRBCs containing healthy single parasites (37.1% ± 3.2 vs. 31.6% ± 5.1, MIIG mice vs. C57BL/6J day 6 p.i., n.s.). Single parasite parasitemia peaked at day 6 in both MIIG and C57BL/6 mice but remained elevated for an additional day in the MIIG mice, while it dropped significantly in the C57BL/6 mice (35.6% ± 5.2 vs. 24.8% ±3.9, MIIG mice vs. C57BL/6J day 7 p.i., *p* = 0.0038). The percent of iRBCs containing multiple parasites was approximately twice that observed in C57BL/6 mice on days 6 and 7 p.i. (14.6% ± 1.9 vs. 6.1% ± 1.6 MIIG mice vs. C57BL/6J, day 6 p.i. *p* < 0.0001 and 12.1% ± 1.9 vs. 7.3% ± 2.0 MIIG mice vs. C57BL/6J, day 7 p.i. *p* = 0.009). By day 8, the percent parasitemia of intact *B. microti* was comparable in both groups of mice. Crisis-form development was significantly elevated at day 7 in C57BL/6 mice compared to MIIG mice (12.7% ± 4.0 vs. 1.1% ± 3.5 C57BL/6 mice vs. MIIG, day 7 p.i. *p* < 0.0001). Crisis-form development peaked on day 8 in C57BL/6 mice and MIIG mice (16.3% ± 3.9 vs. 11.6% ± 3.5 C57BL/6 mice vs. MIIG, day 8 p.i., n.s.) but rapidly decreased in MIIG mice, whereas it remained elevated in C57BL/6 mice until day 10. No differences in the duration of parasitemia were observed. These data demonstrate that activation of macrophages through IFNγ plays a limited role in the regulation of parasitemia levels but is either not involved in the clearance of parasites or adequate compensatory mechanisms exist. This again highlights a limited or redundant role for IFNγ in the response to *B. microti*.

### 3.4. ROS Generation Is Critical for Intraerythrocytic Killing of B. microti Parasites

**Figure 5:**
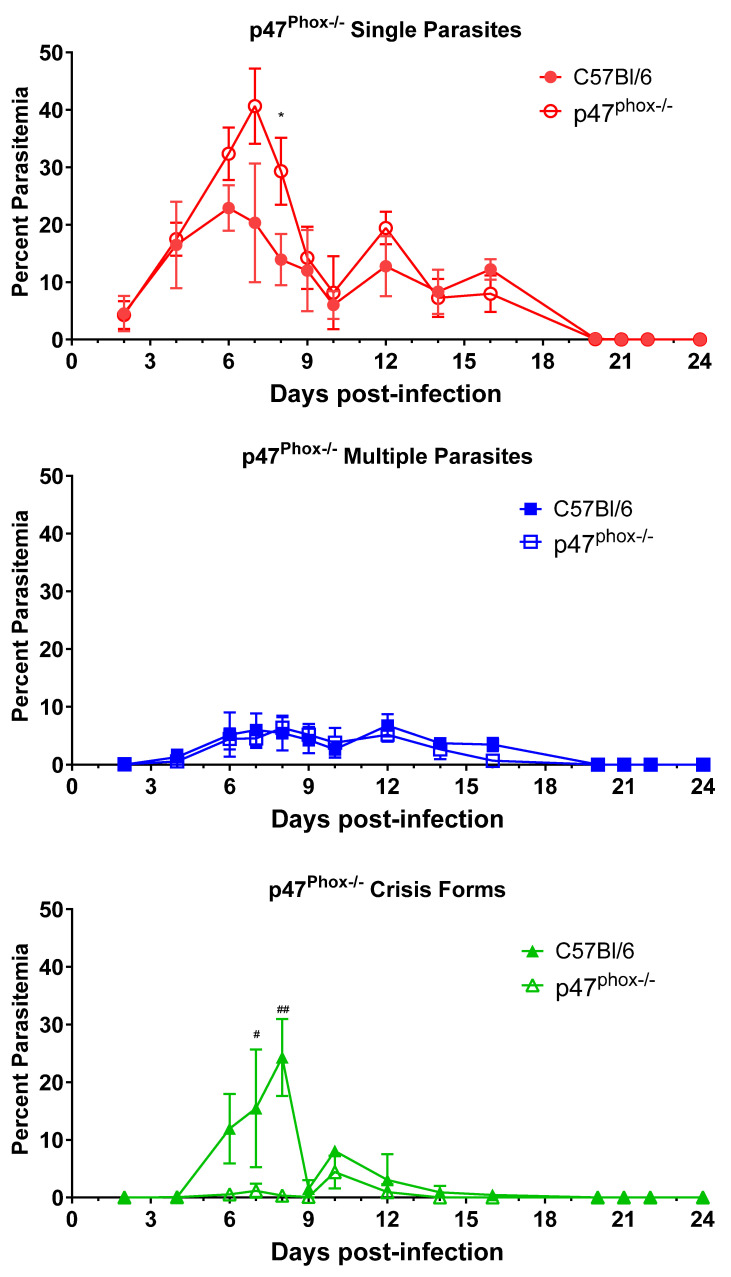
Reduced crisis-form development but not parasite clearance in p47^phox-/-^ mice compared to C57BL/6 mice over the course of infection with *B. microti*. The percentage of RBCs containing single parasites (circles), multiple parasites (squares), or parasites in crisis form (triangles) were enumerated every other day from day 3 post-infection to day 21 post-infection in p47^phox-/-^ mice (closed symbols) and C57BL/6 (intact control, open symbols) mice. Parasite clearance was observed in all mice by day 21. Levels of single, multiple, or crisis-form parasites were compared between p47^phox-/-^ mice and C57BL/6 mice using the multiple *t*-test in PRISM 10; adjusted *p*-values are reported. An *p* < 0.05 was considered significant. * *p* < 0.05 between p47^phox-/-^ mice and C57BL/6 single parasites, # *p* < 0.05, ## *p* < 0.01 between p47^phox-/-^ and C57BL/6 crisis forms, and. *n =* 4–5 mice per group. The C57BL/6 mice in this figure are the same as in Figure 2, as both groups of knock-out mice were infected in parallel and referenced to the same control group.

Intraerythrocytic death of parasites within intact RBCs is an unexplained phenomenon that we observed during the course of infection. We hypothesized that intraerythrocytic death is the result of immune function, as changes in some immune factors have the ability to influence crisis-form development. Others have suggested to us that crisis forms may develop due to nutrient depletion within the cells. Crisis-form development is affected by TNFR1/2 deletion, CCR2 deficiency, and IFNγ activation of macrophages. IFNγ and TNFα activation of phagocytes increases killing through a variety of mechanisms, including reactive oxygen species (ROS) generation. To evaluate the role of ROS in crisis-form development, we infected p47^phox-/-^ mice with *B. microti* (Figure 5). p47^phox-/-^ mice have a defect in the production of superoxide radicals by nicotinamide dinucleotide phosphate (NADPH) oxidase [22]. Apart from a single day, day 10 p.i., crisis forms were present in less than 1.2% of iRBCs in p47^phox-/-^ mice, while in C57BL/6 mice, crisis forms peaked on day 8 p.i. and were significantly higher than p47^phox-/-^ mice on both day 7 and 8 p.i. (Day 7: 17.1% ± 7.6 vs. 1.2 ± 1.2, *p* = 0.04; Day 8: 24.3% ± 6.7. vs. 0.3% ± 0.3, *p* = 0.002 C57BL/6 vs. p47^phox-/-^). Correspondingly, the percent parasitemia of iRBCs containing healthy single parasites was increased in the p47^phox-/-^ mice compared to C57BL/6 mice (29.3 ± 5.8 vs. 13.9 ± 4.5, p47^phox-/-^ vs. C57BL/6, day 8 *p*.i., *p* = 0.03). Single parasite levels were also elevated on days 6 and 7 in p47^phox-/-^ compared to C57BL/6 but failed to reach statistical significance due to a wide standard deviation (*p* = 0.11 for both timepoints). The percentages of iRBCs containing multiple parasites were similar throughout infection. As with CCR2-/- and MIIG mice, p47^phox^ deficiency did not affect parasite clearance. These data strongly suggest the ROS, particularly superoxide radicals produced by NADPH oxidase, play a significant role in the intraerythrocytic death of *B. microti*.

### 3.5. Phosphatidylserine-Mediated Recognition and Clearance of Damaged RBCs by Phagocytes Does Not Play a Role in Clearance of B. microti

The steps leading to the initial recognition of the *B. microti* parasite by the immune system are unclear, as are the mechanisms of clearance of *B. microti* infected RBCs. We tested a hypothesis that *B. microti* parasitization leads to upregulation of phosphatidylserine (PS) on the surface of infected RBCs, which in turn would lead to increased recognition of these cells by splenic and other phagocytes. To test this, mice were administered the D89E mutant of Milk Fat globulin-EGF-factor 8 (MGF-E8), a protein that binds to aminophospholipids such as PS on apoptotic cells and facilitates their uptake by phagocytic cells by interacting with αvβ3 integrins [16]. The D89E mutant binds to PS and other aminophospholipids but does not interact with the integrin, effectively shielding recognition of these cells from PS-mediated clearance mechanisms. To test if upregulation of PS on the surface of iRBCs was involved in the recognition, killing, or clearance of *B. microti* by the immune system, we administered the D89E dominant negative mutant of MGF-E8 to mice as previously described [18]. The D89E mutant had no effect on the course of babesiosis in D89E-administered mice, as compared to controls (Supplementary Figure S1).

## 4. Discussion

The immunological mechanisms responsible for protection from *B. microti* infection have been described only in broad strokes. It is known that CD4+ T cells play an important role in clearing the parasite as CD4-/- mice or mice depleted of CD4+ cells develop a persistent parasitemia. Macrophages are also believed to play an important role in controlling the infection, as depletion of macrophages using chlodronate liposomes (CLL) at various timepoints during infection resulted in increased and prolonged parasitemia and a significant reduction in Th1 cytokines [23]. The importance of the spleen in babesiosis is underscored by the lethality of the disease in splenectomized patients [1], highlighting its likely role as the primary site of immunity against the pathogen. Despite this, studies have yet to identify any other factors that are critical for protection against the parasite. Loss of IFNγ, B cells/antibody, TLR signaling through MyD88, and iNOS 2 production of NO all had limited or no impact on the course of disease in mice [9], indicating they do not play a role or there are multiple layers of redundancy in the immune response. In the present study, we identified TNFR signaling as the first immunological factor that impacted every measure of infection that we evaluated: the magnitude of parasitemia for iRBCs parasitized with single organisms or multiple organisms, the duration of parasitemia, and the intraerythrocytic killing of *B. microti*. TNFR1a and TNFR1b are the receptors for both TNFα and LTα (formerly known as TNFβ) [19,20]. The model system we utilized cannot distinguish between roles for TNFα and LTα; however, this kind of activity would be an unprecedented role for LTα. These results strongly suggest that TNFα plays a critical role in the response to *B. microti*. Parasitized RBCs were detected for as long as 36 days in the absence of TNFR. Blood smears were not fully evaluated until after the experiment was terminated, resulting in premature termination. Parasitemia levels were trending towards ultimate clearance; however, indicating that redundant mechanisms in the immune response are present to provide protection in the loss of TNFα signaling. These mechanisms can include IFNγ and antibodies, which, in their own, are not critical for protection against *B. microti* but may become critically important in the absence of normal immune function. Experiments to further characterize the role of TNFα and potential protective redundancies are being designed now.

Little is known about the significance of different population structures of *B. microti* during mammalian infection. Significant differences in parasite morphologies are observed; RBCs are infected with either single parasites, multiple parasites, or parasites in crisis form. Crisis forms have been shown to be dead or dying parasites within erythrocytes. However, the significance of multiply infected vs. singly infected RBCs is unclear. A related parasite, *B. divergens*, builds a complex population structure that allows the parasite to rapidly respond to changing environmental conditions in vitro in RBC culture. In the case of *B. divergens*, multiple parasites within an iRBC are thought to result from a checkpoint that induces parasite replication within the cell with lack of egress, rather than a single cell being infected with multiple independent parasite invasion events [24]. If *B. microti* has a similar population structure, the bias toward multiple parasites within an iRBC would represent a pool of parasites that accumulates during stressful growth conditions, such as observed in our TNFR1/2 KO mice. These could be rapidly released to reseed multiple RBCs to sustain parasite persistence. It is our hypothesis that the accumulation of multiple *B. microti* parasites within RBCs suggests an environment that is more stressful to the parasite than when single parasites predominate within RBCs without affecting parasite viability, which would result in crisis-form development. Thus, healthy parasites multiply in the iRBC but delay egress until more favorable conditions present, allowing for continued infection.

Parasite persistence is a serious problem for individuals with babesiosis, both in terms of symptomatic and asymptomatic infections [6]. In studies of blood donors, individuals who were asymptomatic have been shown to have viable parasites, including protracted low and intermittent levels of parasitemia that could last over two years. The underlying mechanisms that contribute to long-term persistence in some apparently immune-competent individuals are unknown. However, individuals with deficits in B cell immunity often due to targeted immunotherapy are at high risk of severe disease and long-term carriage, pointing to critical roles for both B cells and generalized immune suppression in disease severity and parasite persistence [6]. Our data suggest that an impaired cytokine response, including TNFα or LTα, may also contribute to parasite persistence, but more data in humans with babesiosis and polymorphisms that impact TNFα are needed.

In addition to TNFR signaling, the role of macrophages was investigated more thoroughly. Tissue-resident macrophages are typically generated from tissue-localized precursors [25]. During inflammatory responses, inflammatory monocytes are recruited to the site of inflammation in a CCR2-dependent manner [21]. Elimination of the axis in CCR2-/- mice had no effect on parasite clearance but did limit the development of crisis-form parasites in iRBCs, resulting in a corresponding increase in iRBCs parasitized by healthy *B. microti*. This could indicate a limited role of inflammatory monocytes in the response; however, the spleens of mice contain their own pool of inflammatory monocytes [26], in addition to splenic red pulp macrophages, which may have limited the impact of CCR2 deficiency on the observed response. In addition, we investigated the importance of the IFNγ–macrophage axis in parasite clearance by utilizing mice whose macrophages express a dominant negative IFNγ receptor incapable of signaling. Thus, the macrophages of MIIG mice cannot respond to IFNγ stimulation. They displayed an interesting phenotype in which iRBCs parasitized with multiple parasites were present at a level almost twice that observed in wild-type mice during peak parasitemia. Peak numbers of crisis forms were similar between both groups of mice but rapidly decreased in MIIG mice. Thus, lack of activation of macrophages by IFNγ did impact parasitemia in a limited way, but compensatory mechanisms, potentially through TNFα, led to clearance of the parasite.

We previously demonstrated that parasite death and clearance of iRBCs did not involve iNOS2-mediated production of nitrogen radicals [9]. Limited transcriptomic data that we have generated (manuscript in preparation) suggest that the parasite is upregulating defense mechanisms against oxidative stress. We therefore investigated whether loss of NADPH oxidase-derived superoxide radicals would impact the course of disease. P47^phox-/-^ mice displayed a significant reduction in the development of crisis forms within iRBCs, with a corresponding increase in healthy single parasites within iRBCs but not multiple parasites. These results indicate that superoxide production by NADPH oxidase, a major constituent of respiratory burst by immune cells, plays a key role in the intraerythrocytic death of parasites. It had been previously suggested to us that parasite death was being caused by limited nutritional resources within iRBCs. In total, our results strongly suggest that parasites are being killed within intact RBCs through an unknown immune-mediated mechanism that includes NADPH oxidase. We are currently designing experiments to assess if there is a link between CCR2+ cells and NADPH oxidase-mediated generation of crisis forms.

Finally, it is unclear how the immune system first identifies the presence of *B. microti*. After the tick bite, the parasite rapidly enters RBCs in the blood stream. This should provide nominal protection from immune recognition, and we hypothesize that recognition occurs first through clearance of iRBCs by the reticuloendothelial system. One mechanism by which this is carried out is through the recognition of PS on the surface of damaged or senescent RBCs. We used a dominant negative mutant of MGF-E8 to coat PS on the surface of senescent cells and prevent their recognition by phagocytes. This had no observable effect on the course of babesiosis, indicating that it does not play a role in the detection of iRBCs.

Some limitations of this study are that we utilized needle inoculation of mice rather than natural tick-borne transmission. Therefore, we cannot anticipate how the immune changes we observe here might be affected by factors in natural tick transmission, such as the influence of tick salivary proteins. In addition, rodents, though not standard laboratory mice, are part of the normal lifecycle of the parasite. While useful as a model system, the mouse does not reproduce some of the symptomatic features of human disease, including severe complications of babesiosis, and the infection is rarely fatal to mice in the absence of a severe immune compromised state.

*B. microti*, as the causative agent of human babesiosis, can cause severe disease requiring hospitalization and can cause death. Both the geographical range of the disease and incidence are increasing, leading to a higher incidence of severe complications. Understanding the immunological mechanisms that underlie protection from infection is paramount to understanding why some individuals develop severe complications, while others can have a relatively mild infection or remain asymptomatic. Here, we show for the first time, to our knowledge, that TNFR signaling, likely through TNFα engagement, is a critical host-derived factor that plays a role in the killing of parasites and the clearance of iRBCs. As the factors that define protective immunity become clear, we can begin to identify and mitigate deficiencies that predispose some individuals to severe infection.

## Data Availability

The dataset is available upon request from the authors.

## Supplementary Materials

The following supporting information can be downloaded at: https://www.mdpi.com/article/10.3390/pathogens13100858/s1. Figure S1: Blocking of phosphatidylserine-mediated clearance of senescent and damaged cells does not affect parasitemia. Mice were administered D89E by IV injection beginning the day prior to infection with *B. microti*. D89E administered animals (closed symbols) had the same course of parasitemia as untreated animals (open symbols) for both healthy (circles) and crisis-form (triangles) parasites. Single and multiple parasites were not differentiated in this experiment. *n* = 9–10 mice per group.
