# Supplementary figures and images for: Tumor Necrosis Factor Receptors and C-C Chemokine Receptor-2 Positive Cells Play an Important Role in the Intraerythrocytic Death and Clearance of Babesia microti
Source: Pathogens. 2024 Oct 1;13(10):858. doi: 10.3390/pathogens13100858 (PMC11510159; doi:10.3390/pathogens13100858)

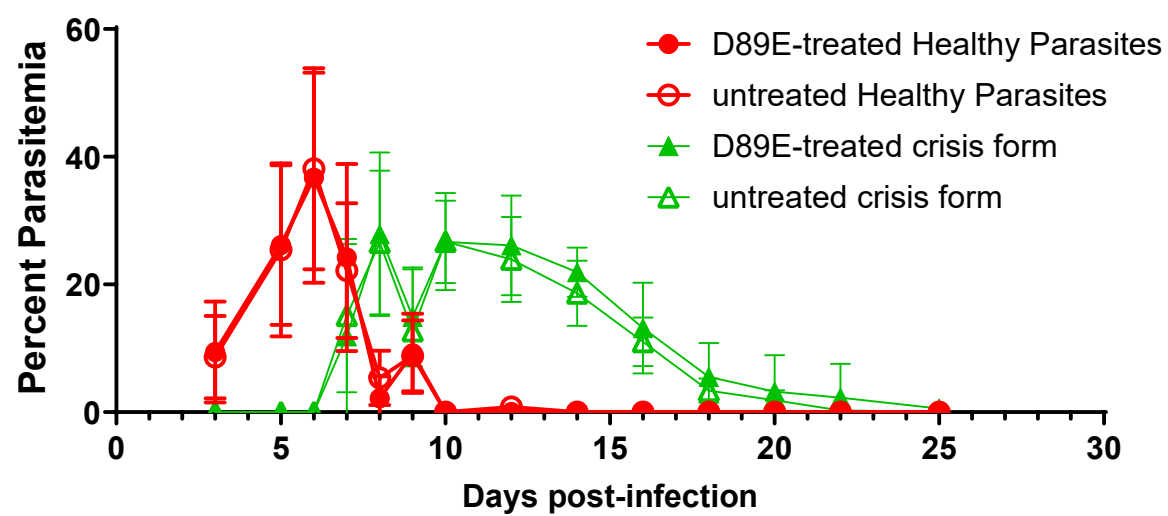

Supplement: Supplementary file 1 [file pathogens-13-00858-s001.zip › pathogens-3218247-supplementary.pdf]
